# Heparin-binding enhances extracellular listeriolysin O activity, overcoming cholesterol inhibition and pH dependence

**DOI:** 10.1128/jb.00526-25

**Published:** 2026-06-12

**Authors:** Luigi La Pietra, Helena Pillich, Martina Hudel, Besim Berisha, Mobarak Abu Mraheil, Andreas Geissner, Peter H. Seeberger, Trinad Chakraborty

**Affiliations:** 1 Institute of Medical Microbiology, Justus Liebig University Giessen, Giessen, German Center for Infection Research (DZIF), Partner site Giessen-Marburg-Langen 9175, Giessen, Germany; 2 Biomolecular Systems Department, Max Planck Institute of Colloids and Interfaces, Potsdam, Germany; Indian Institute of Technology Bombay, Mumbai, Maharashtra, India

**Keywords:** *Listeria monocytogenes*, heparin-binding proteins, listeriolysin (LLO)-heparin interaction, LLO purification, D4 domain, core ligand, heparan sulfate

## Abstract

The membranolytic toxin listeriolysin O (LLO), a member of the family of bacterial cholesterol-dependent cytolysins (CDC), is a key virulence factor of *Listeria monocytogenes* (Lm). Current evidence indicates that LLO is primarily a unique site-specific intracellular CDC, as it is highly active at the acidified pH of phagosomes but exhibits reduced activity and is prone to irreversible denaturation at physiological pH. Here, we demonstrate that LLO binds heparin and exploit this finding to affinity-purify the toxin in a single step. Heparin-binding increases LLO hemolytic activity at neutral pH manifold, and further amplifies the increased activity conferred by treatment with the thiol-reducing reagent DTT. Heparin-activated LLO is insensitive to inhibition by cholesterol, and its activity is not pH-dependent. We localized heparin binding to the C-terminal D4 membrane-interacting domain of LLO and used glycan arrays to define the minimal sulfated ligand engaged. Molecular docking studies suggest a heparin-binding site within this domain, and a LLO variant carrying a mutation (N508A) in this region no longer exhibited heparin-enhancing hemolytic activity. Engaging heparin enhances hemolysis and provides an additional layer of regulation of toxin function that ensures its optimal activity at physiological pH. As LLO is known to promote bacterial entry, and the sulfated heparin substructure recognized is present in many members of the family of cell surface heparan sulfates, our data suggest that this targeted tropism could contribute to the transition of the bacterium from extracellular compartments to intracellular niches.

**IMPORTANCE:**

Bacterial pore-forming cytolysins act extracellularly to disrupt host cell membranes. Listeriolysin (LLO), a cholesterol-dependent cytolysin (CDC) and a key virulence factor of *Listeria monocytogenes*, is distinguished by its site-specific intracellular activity, exhibiting maximal function at the acidic pH of phagosomes where the bacteria reside after uptake. At physiological pH and temperature, LLO exhibits reduced activity and susceptibility to denaturation. Here, we show that LLO binds heparin, a property that greatly enhances its hemolytic activity at neutral pH and overcomes cholesterol-dependent inhibition. We identified both the heparin binding site in LLO and the structure of the minimal interacting ligand engaged. The affinity of LLO for heparin enabled direct purification of highly active toxin from diverse naturally occurring isolates in a single step.

## INTRODUCTION

Listeriolysin O (LLO), a cholesterol-dependent cytolysin (CDC) and member of the class of β-pore forming toxins (PFTs), is an essential virulence factor of the human food pathogen *Listeria monocytogenes* (1). LLO is the only known CDC member of the family of pore-forming toxins that is expressed by intracellular bacteria (2). Unlike other extracellular CDCs such as streptolysin and perfringolysin, whose cytolytic activities are similar at both pH 5.5 and 7.4, LLO activity is uniquely optimal at pH 5.5. Replacing LLO with CDCs whose activities are pH-independent resulted in strains that can escape from the phagosome but kill the infected host cell, due to their overt toxicity, thereby eliminating the intracellular replicative niche (2, 3). This pH preference is seen as a property that has been strongly influenced by the adaptation of this bacterium to an intracellular lifestyle (2, 4, 5).

LLO is considered a versatile virulence factor of pathogenic *L. monocytogenes* because it is involved in many important processes ranging from facilitating internalization of the bacterium into host cells to promoting intracellular survival and enabling cell-to-cell spread (2, 4, 6). It activates the host cell endocytic machinery or phagocytosis to promote bacterial entry into the host cell (7, 8). A major function of LLO is its membranolytic activity, a function required for the release of bacteria from primary vacuoles following entry into host cells as well as during cell-to-cell spread (9). LLO is also required for resolving the intercellular protrusion into a vacuole by damage to the double membrane vesicle, triggering the recipient cell to internalize the bacterium (10). Beyond its canonical role as a pore-forming toxin, LLO functions as a potent modulator of host cell functions, triggering complex signaling cascades that influence inflammation, autophagy, and calcium homeostasis (2, 4, 7).

Structural features within LLO enable the modulation of its intracellular activity both within vacuolar compartments and the host cytosol and underlie its ability to promote bacterial growth by avoiding overt destruction of this replicative niche (5). Major mechanisms that reduce intracellular LLO activity include ubiquitination of its N-terminal lysine, particularly in combination with the PEST sequence, or by S-glutathionylation of its C-terminal cysteine residue. While the former mechanism promotes intracellular degradation of LLO, the latter modification leads to loss of its cytolytic ability (5, 11, 12). An interaction between the PEST sequence with the Ap2a2 subunit of the host clathrin-dependent endocytosis machinery accelerates uptake of membrane-associated LLO by endocytosis into autophagosomes, thereby preventing cytotoxicity (13). LLO can also be cleaved by the phagosomal protease cathepsin D (14) or degraded by the neutrophil metalloproteinase-8 (15). Furthermore, the hemolytic activity of LLO is also strongly inhibited by plasma membrane components such as cholesterol, phosphocholine, and the C-series of gangliosides (16–18). Both thiol reduction by the gamma-interferon-inducible lysosomal thiol reductase (GILT) and vacuolar acidification by the cystic fibrosis transmembrane conductance regulator (CFTR) promote toxin activity in phagosomes and facilitate bacterial egress (19, 20).

LLO is synthesized as a water-soluble monomer and is secreted by *L. monocytogenes* (3, 8, 16). The crystal structure of LLO reveals a modular organization comprising four major domains (D1-4) arranged around a central axis (21). Domain D4 contains the primary binding site for cholesterol and comprises a highly conserved undecapeptide essential for initiating membrane interaction. The remaining domains (D1, D2, and D3) and their flexible linkers are critical for orchestrating the subsequent steps of oligomerization and pore formation (21, 22). For many years, LLO and other CDC toxins were characterized by their ability to bind cholesterol, which is generally considered a CDC receptor (18, 22). Cholesterol does not interfere with LLO’s ability to bind to the host cell membrane or the ability to induce cytokine expression in macrophages, suggesting the presence of additional host factors promoting its uptake (16, 23). Notably, LLO binds to archaeal lipid vesicles from *Aeropyrum pernix* K1 that lack cholesterol. As these membranes contain carbohydrate inositol in their head group, it was postulated that LLO can use carbohydrates for binding (24). Affinity-based studies using glycan arrays have indeed revealed specific and distinct lectin-like properties for various members of the family of CDC toxins and demonstrated that LLO binds to carbohydrate moieties found on the C-series gangliosides (25). Interactions between these ganglioside structures and LLO were pH-dependent and resulted in an inhibition of its hemolytic activity. As with cholesterol, this activity was located within the C-terminal D4 domain of LLO, whose structural tip is needed for the initial membrane contact, leading to pore formation (21).

The hemolytic property of LLO serves as a proxy indicator to monitor pathogenic *L. monocytogenes* strains in clinical and food samples (26). Production of LLO *by L. monocytogenes* is highly regulated by environmental factors such as growth temperature, the availability of nutrients such as sugars, branched-chain amino acids, and thiol oxidoreductase-dependent post-translational modification of the positive-acting regulator protein of virulence PrfA, which operates dedicated transcriptional control mechanisms to control LLO expression (12, 27–33).

It has been suggested previously that the soluble-sulfated glycosaminoglycan heparin binds proteins on the surface of *L. monocytogenes* and plays a role in extraintestinal dissemination of this enteric bacterium (34). Pretreatment of *L. monocytogenes* with heparin inhibited its adhesion and entry into both phagocytic and non-phagocytic cell lines (35). Subsequent studies implicated its surface-bound actin interaction protein ActA as the ligand for host cell surface heparan sulfates (HS) (36). Another surface-bound listerial protein, internalin B (InlB), which engages the hepatocyte-growth factor on host cells, also was found to require mammalian surface HS for promoting bacterial entry into host cells. Studies using InlB mutant variants in combination with a CHO (Chinese Hamster Ovary) cell line deficient in HS indicated that HS binding promotes both InlB-dependent bacterial adherence and invasion (37). More recently, a major family of the HS-containing proteoglycan Syndecan-1 (Sdc1) was found to promote listeriosis (38).

We examined supernatant fractions of *L. monocytogenes* for secreted proteins that bind the HS-mimic, heparin. We detected LLO as a major secreted protein species with specific heparin binding properties and exploited this finding to develop a simple procedure to purify this PFT from different *L. monocytogenes* isolates. As sulfated glycosaminoglycans occur primarily on host-cell surfaces, we examined the effects of this binding on the hemolytic activity of LLO. We find that LLO-dependent heparin binding increases toxin hemolytic activity manifold and protects it from cholesterol- and phosphocholine-dependent inhibition. We localize heparin-binding to the compact D4 domain of LLO and define both a minimal ligand structure that is recognized by the toxin as well as those residues required for this interaction.

## RESULTS

### Identification of listeriolysin O as a heparin-binding component in the supernatant fluids of *L. monocytogenes*

**Fig 1:**
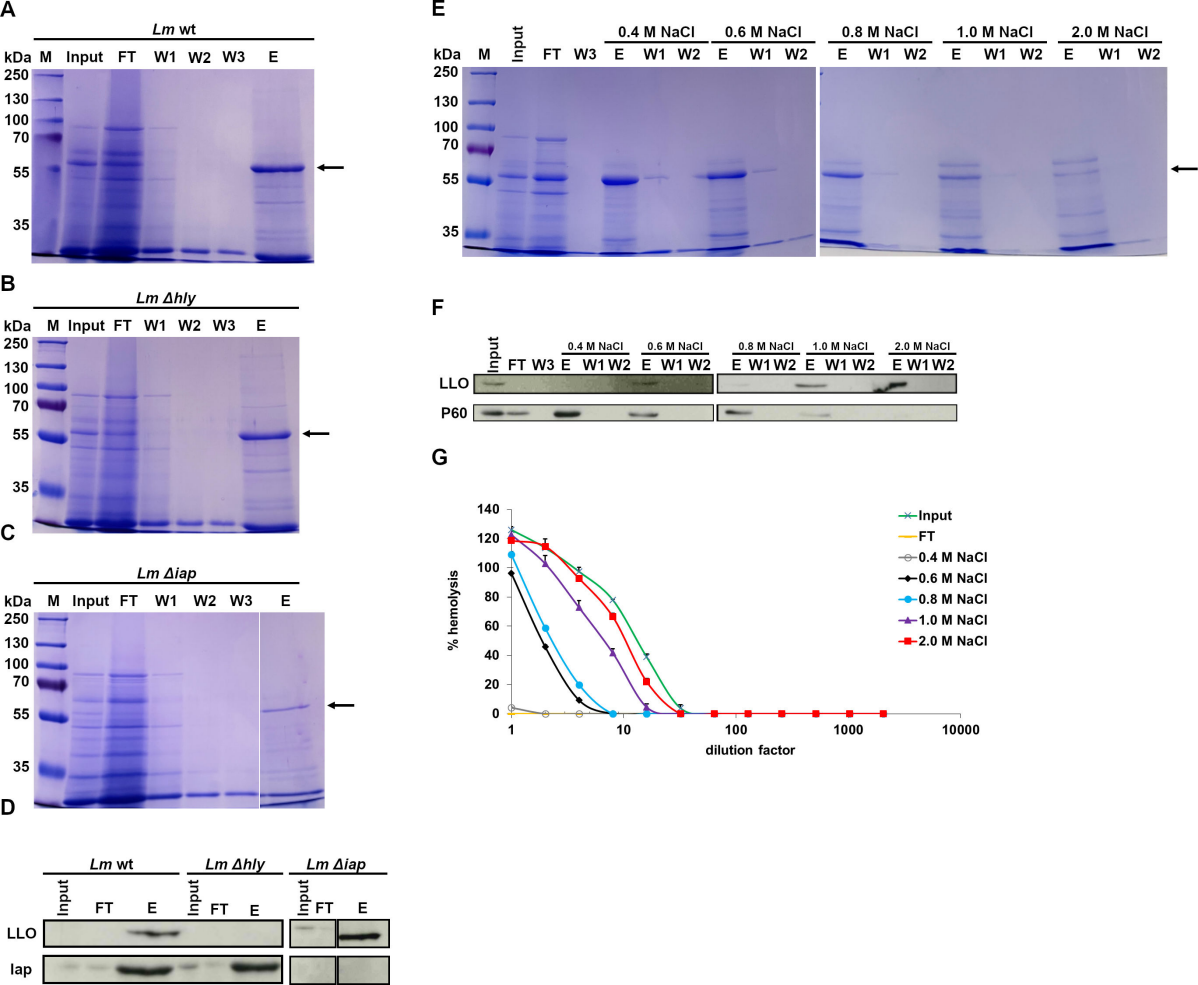
Elution profile of concentrated crude supernatant of *L. monocytogenes* (*Lm*) EGD-e (**A**) or *Lm* EGD-e Δ*hly* (**B**) and *Lm* EGD-e Δ*iap* (**C**) binding to heparin sepharose beads in batch format. The arrow indicates an eluted protein band with elution buffer containing 2 M NaCl between 58 and 60 kDa. M = marker; Input = concentrated supernatant (25 µL); FT = flow through (50 µL); W1–W3 = wash fractions (50 µL), and E = elution fraction (10 µL). (**D**) Immunoblot analysis of input, FT, and elution fraction from the above shown fractions. An anti-LLO and an anti-Iap antibody were used for the detection of LLO and Iap, respectively. (**E**) Elution profile of concentrated crude supernatant of *L. monocytogenes* EGD-e binding to heparin sepharose beads in batch format. The arrow indicates a protein band containing either LLO and/or Iap. Washing steps were performed with the binding buffer. Elution buffer with indicated sodium salt concentrations was used to obtain elution fractions. M = marker; Input = concentrated supernatant (10 µL); FT = flow through (50 µL); W1–W3 = wash fractions (50 µL); and E = elution fraction (30 µL). (**F**) Immunoblot analysis of input, FT, wash fractions, and elution fraction from the above shown fractions. An anti-LLO and an anti-Iap antibody were used for the detection. (**G**) Sheep erythrocytes were incubated with 5 µL of indicated collected elution fractions (diluted 1:5) at 37°C for 1 h. After centrifugation, the optical density (OD) at a wavelength of 405 nm (OD_405 nm_) was measured. The elution fractions eluted with 1.0 M NaCl and 2.0 M NaCl (purple triangles; red squares) showed the highest hemolytic activity, which was comparable to the hemolytic activity of the input (green crosses). No hemolytic activity was detectable in the FT (yellow bars) and the elution fraction with 0.4 M NaCl (gray small circles). Elution fractions eluted with 0.6 M NaCl (black diamonds) and 0.8 M NaCl (blue circles) showed moderate hemolytic activity. Mean values ± SEM are plotted from three independent experiments.

Sterile-filtered culture supernatant fluids obtained from growing cultures of *L. monocytogenes* EGD-e were examined for protein binding after incubation with heparin sepharose beads, followed by SDS-PAGE analysis and immunoblotting (see Methods section). Incubated beads were washed three times with the incubation buffer, and the bound proteins were eluted with the elution buffer. SDS-PAGE indicated the presence of two major polypeptide species at 60 kDa and 25 kDa, respectively (Fig. 1A). Previous studies have indicated that polypeptides present in spent supernatants of *L. monocytogenes* of between 58 and 60 kDa comprise two abundant proteins, the autolysin p60 (also known as invasion-associated protein [Iap]) and secreted LLO (39, 40). Elution profiles from supernatants from *L. monocytogenes* Δ*hly*, the LLO deletion mutant, and *L. monocytogenes* Δ*iap* confirmed that both proteins are bound and eluted from these affinity columns (Fig. 1B and C). This was further confirmed by using specific mAbs directed toward detecting Iap and LLO (Fig. 1D).

We examined the profiles of polypeptides following elution with buffers containing increasing concentrations of NaCl using SDS-PAGE and immunoblotting with specific anti-Iap and anti-LLO antibodies. We found that Iap was abundant in fractions eluting in early fractions up to 0.8 M NaCl, while LLO was present in eluates of much higher salt concentrations, viz. 1 M and 2 M NaCl (Fig. 1E and F). This was confirmed by determining hemolytic activities in the various elution fractions. The highest hemolytic activity was present in the fractions eluted with 1 M and 2 M NaCl (Fig. 1G).

### Single-step heparin sepharose resin-based affinity purification of listeriolysin O

**Fig 2:**
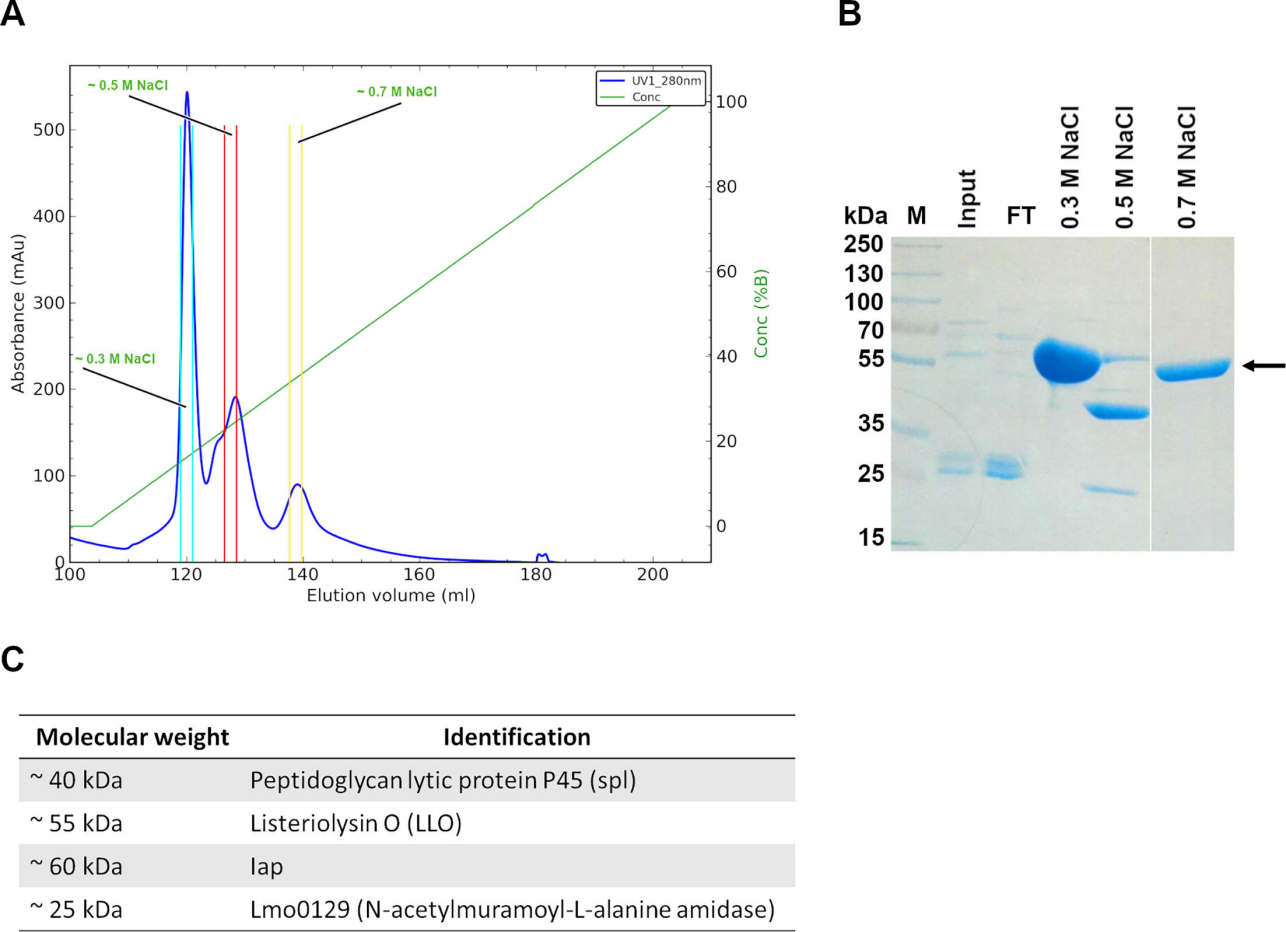
(**A**) FPLC chromatogram output from HiTrap heparin affinity column of supernatant from *L. monocytogenes* EGD-e. Colored bars indicate selected elution fractions. Bound proteins were eluted with a linear gradient of elution buffer (0–2 M NaCl). Proteins eluted as three major peaks at salt concentrations of 0.3 M NaCl (blue), 0.5 M NaCl (red), and 0.7 M NaCl (yellow). Flow rate was at 4 mL/min, and elution fractions were collected every 50 s. mAU indicates absorption at 260 nm. (**B**) Coomassie Brilliant Blue staining of the protein isolation. Shown is the input, flow through (FT), elution fraction at 0.3 M NaCl, at 0.5 M NaCl, and at 0.7 M NaCl. The arrow indicates the protein band corresponding to LLO. M = Marker; Input = concentrated supernatant (30 µL); FT (30 µL); 0.3 M NaCl to 0.7 M NaCl = elution fractions (50 µL). (**C**) The results of the protein identification by mass spectrometry and database search.

These preliminary experiments suggested that heparin-based affinity chromatography could be used for targeted purification of LLO directly from spent culture supernatants. Concentrated crude bacteria-free supernatants of *L. monocytogenes* EGD-e were loaded onto a sepharose-based heparin affinity column, and the proteins that bound to resin were eluted by a linear gradient of elution buffer (Fig. 2A). In the fractions collected, we obtained clear separation of the Iap autolysin and LLO, which eluted at 0.3 M and >0.7 M NaCl, respectively (Fig. 2B). Hemolytic activity was present only in the fractions eluting at >0.7 M NaCl (Fig. S1). The identities of the various protein species seen following SDS-PAGE were determined using MALDI-TOF. The 60 kDa protein eluting at 0.3 M NaCl was confirmed to be the autolysin Iap, while the 58 kDa polypeptide that eluted at 0.7 M NaCl was identified as LLO. We also subjected the protein bands seen at 40 kDa and 25 kDa to further analysis and found that the polypeptide at ~40 kDa corresponded to another listerial autolysin designated Spl, while the sequence of the 25 kDa protein corresponded to the polypeptide encoded by *L. monocytogenes* 0129 (Lmo0129), annotated as an N-acetylmuramoyl-L-alanine amidase encoded by a cryptic listerial bacteriophage (Fig. 2C).

We extended our demonstration of a single-step purification procedure for LLO using the heparin-based strategy by examining elution profiles from supernatants of other *L. monocytogenes* isolates of differing serotypes that expressed varying levels of the toxin, i.e., SLCC2755 serotype 1/2b, serotype SLCC2372 1/2c, and L99 serotype 4a. Elution profiles were very similar to those obtained for the prototype serotype 1/2a isolate (Fig. S2A through C). In all cases, fractions eluting at ~1 M NaCl consisted almost exclusively of purified LLO, whereas Iap was found in fractions eluted at ~0.6 M NaCl. We wondered whether the heparin-binding properties of LLO also extended to another member of the CDC family of toxins, pneumolysin (PLY) of *Streptococcus pneumoniae*. Affinity chromatography using the same heparin-based approach allowed for single-step purification of PLY from supernatant fluids of a recombinant *Listeria innocua* strain that has been engineered to secrete PLY (see Material and Methods). Unlike LLO, PLY eluted at lower salt concentrations (0.4 M NaCl) (Fig. S4A). We calculated the concentration of LLO purified using heparin-based affinity chromatography and found that yields were between 26 and 320 µg/L with a total activity of between 0.2 and 196 fmol hemolytic units (HU) (Table S1).

### Heparin enhances listeriolysin O-dependent hemolytic activity and protects it from inhibition by cholesterol and phosphocholine

We considered that negatively charged host components such as DNA, RNA, tRNA, and heparin binding could interfere with the hemolytic properties of LLO and monitored for changes of purified LLO both in their presence and absence. Only the addition of heparin enhanced the activity of LLO ~10-fold, irrespective of the source viz. *L. monocytogenes* isolates from which it was purified (Fig. S3A through E). To verify that this is an intrinsic property of LLO and does not simply result from the presence of undetected components present from the heparin-based affinity-purification scheme, we examined for changes in its activity using toxin from the *L. monocytogenes* EGD-e strain purified using ion-exchange chromatography (17). These results show that the addition of heparin to purified toxin increases its activity by 6-fold to 8-fold (Fig. S3F).

This heparin-enhancing effect can be further improved by the addition of reducing agent DTT and is distinct from the combined activity obtained with these reagents. We found that the hemolytic activity of LLO increased 10-fold and 16-fold, respectively, when treated individually either with heparin or DTT. The combined effect of heparin and DTT addition increased LLO activity 186-fold (Fig. S5A). Using a LLO variant (41), in which the invariant cysteine at position 484 has been mutated to serine and does not exhibit DTT-activatable activity, we found that the addition of only heparin increased its activity 12-fold, but did not change when only DTT was added (Fig. S5A). The combined effects of heparin and DTT increased LLOC484S activity by only 8-fold. We also examined the effect of heparin activation in a LLO variant lacking its PEST-sequence, a structural element anchoring the toxin to clathrin-dependent endocytosis machinery, and found that the hemolytic activity of LLO ΔPEST is also activated by heparin (Fig. S5B).

**Fig 3:**
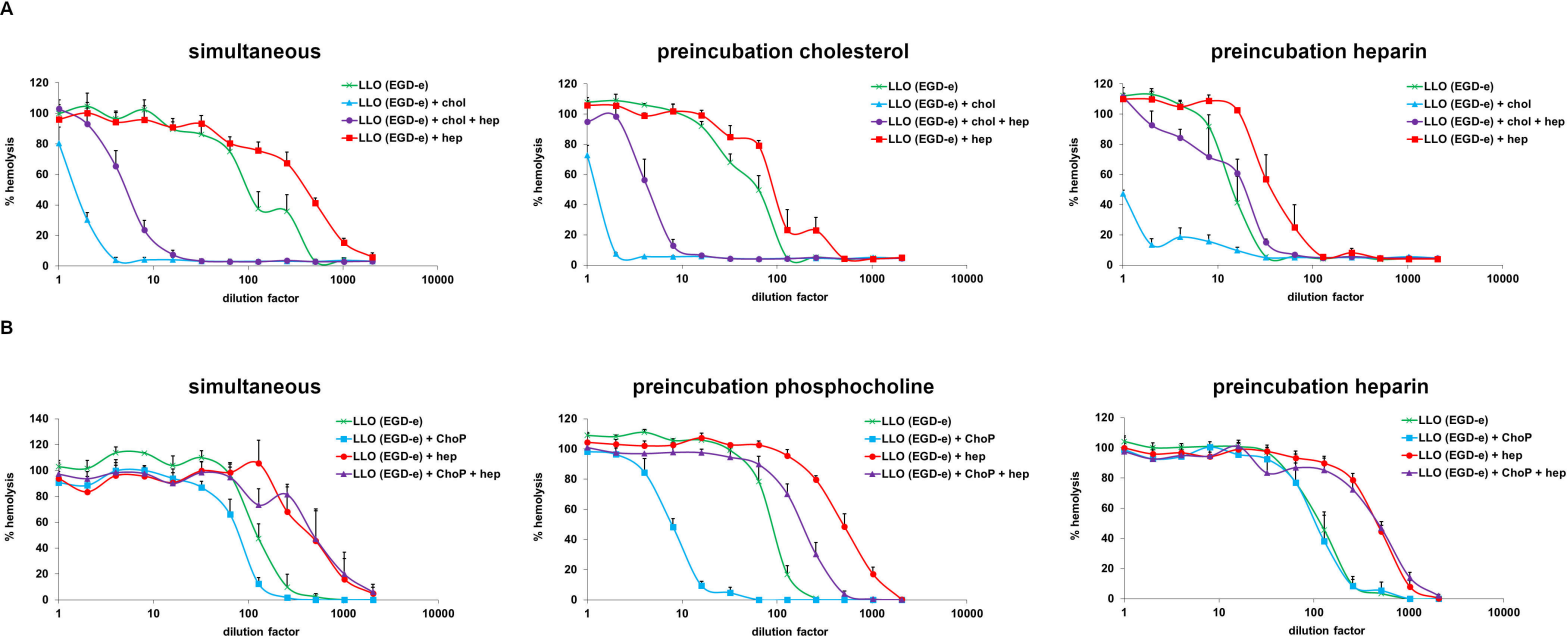
(**A and B**) LLO from *L. monocytogenes* EGD-e (2 μg/mL) was treated simultaneously or pretreated with heparin (hep, 1 µg/mL), cholesterol (chol, 1 µg), or phosphocholine (ChoP, 3.6 mM) and incubated with sheep erythrocytes at 37°C for 1 h. After centrifugation, the optical density (OD) at a wavelength of 405 nm (OD_405 nm_) was measured. Hemolytic activity is strongly inhibited in the presence of cholesterol or phosphocholine and increased in the presence of heparin. Co-treatment of heparin with cholesterol/phosphocholine recovers the hemolytic activity of LLO. Mean values ± SEM are plotted from three independent experiments.

The hemolytic activity of LLO is inhibited by components of the plasma membrane viz. cholesterol and phosphocholine (16, 17). We monitored changes in hemolysis following either simultaneous addition of the inhibitory component together with heparin (hep) or preincubation with either cholesterol (chol) or phosphocholine (ChoP), followed by heparin, and finally, preincubation with heparin prior to the addition of cholesterol or phosphocholine. Overall, heparin has an ameliorating effect on cholesterol-inhibited LLO activity under conditions either when added simultaneously or following pre-incubation of cholesterol (Fig. 3A). Strikingly, preincubation with heparin essentially protects LLO from inhibition by cholesterol. Similarly, the addition of heparin either before or during the addition of phosphocholine restores LLO activity and is even refractory to the subsequent addition of ChoP following prior incubation with heparin (Fig. 3B).

**Fig 4:**
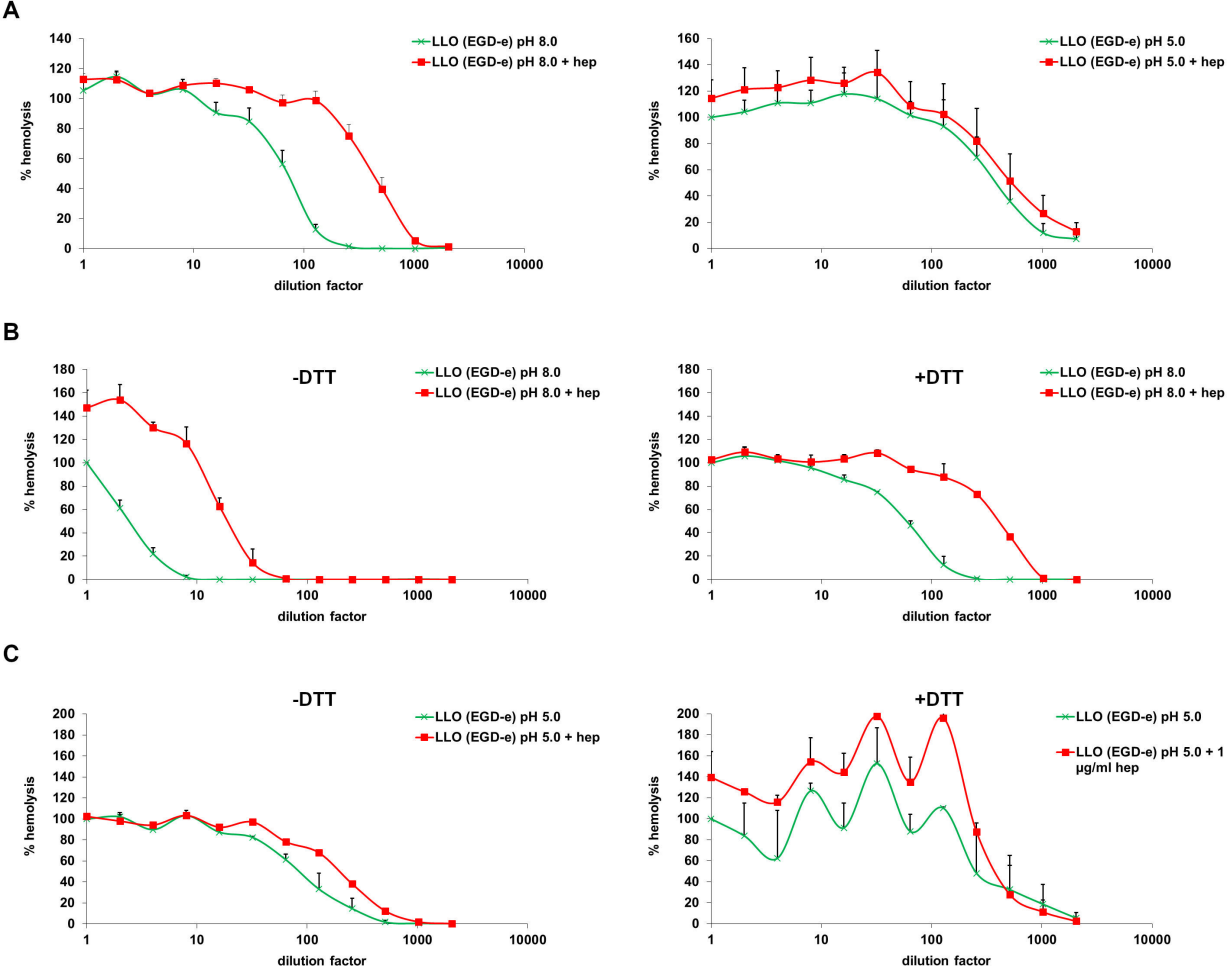
(**A**) LLO from *L. monocytogenes* EGD-e (2 μg/mL) was treated simultaneously with heparin (hep, 1 µg/mL) at pH 8 and pH 5 and incubated with sheep erythrocytes at 37°C for 1 h. After centrifugation, the optical density (OD) at a wavelength of 405 nm (OD_405 nm_) was measured. Hemolytic activity is increased in the presence of heparin at pH 8, but not at pH 5. Shown is one experiment, each repeated twice. (**B and C**) LLO (2 μg/mL) was treated simultaneously with heparin (hep, 1 µg/mL) at pH 8 and pH 5 under nonreducing (−DTT) or reducing (+DTT) conditions and incubated with sheep erythrocytes at 37°C for 1 h. Increase in hemolytic activity in the presence of heparin at pH 8 is not altered under non-reducing conditions. Changes in hemolytic activity in the presence of heparin at pH 5 are unaltered independently of the redox status. Mean values ± SEM are plotted from three independent experiments.

The hemolytic activity of LLO is unique among the family of CDC toxins as its activity is highest at low pH, a condition found within the maturating phagosome (28). We examined the cumulative effects of pH and heparin on LLO-dependent hemolytic activity in buffers at different pH. In assays performed at pH 8.0, heparin enhanced the hemolytic activity more than 10-fold. At pH 5.0, LLO activity is around 10-fold higher than at pH 8.0 and is not enhanced in the presence of heparin (Fig. 4A). This observation was independent of its activation status by DTT (Fig. 4B and C).

### The D4 domain of LLO binds heparin

**Fig 5:**
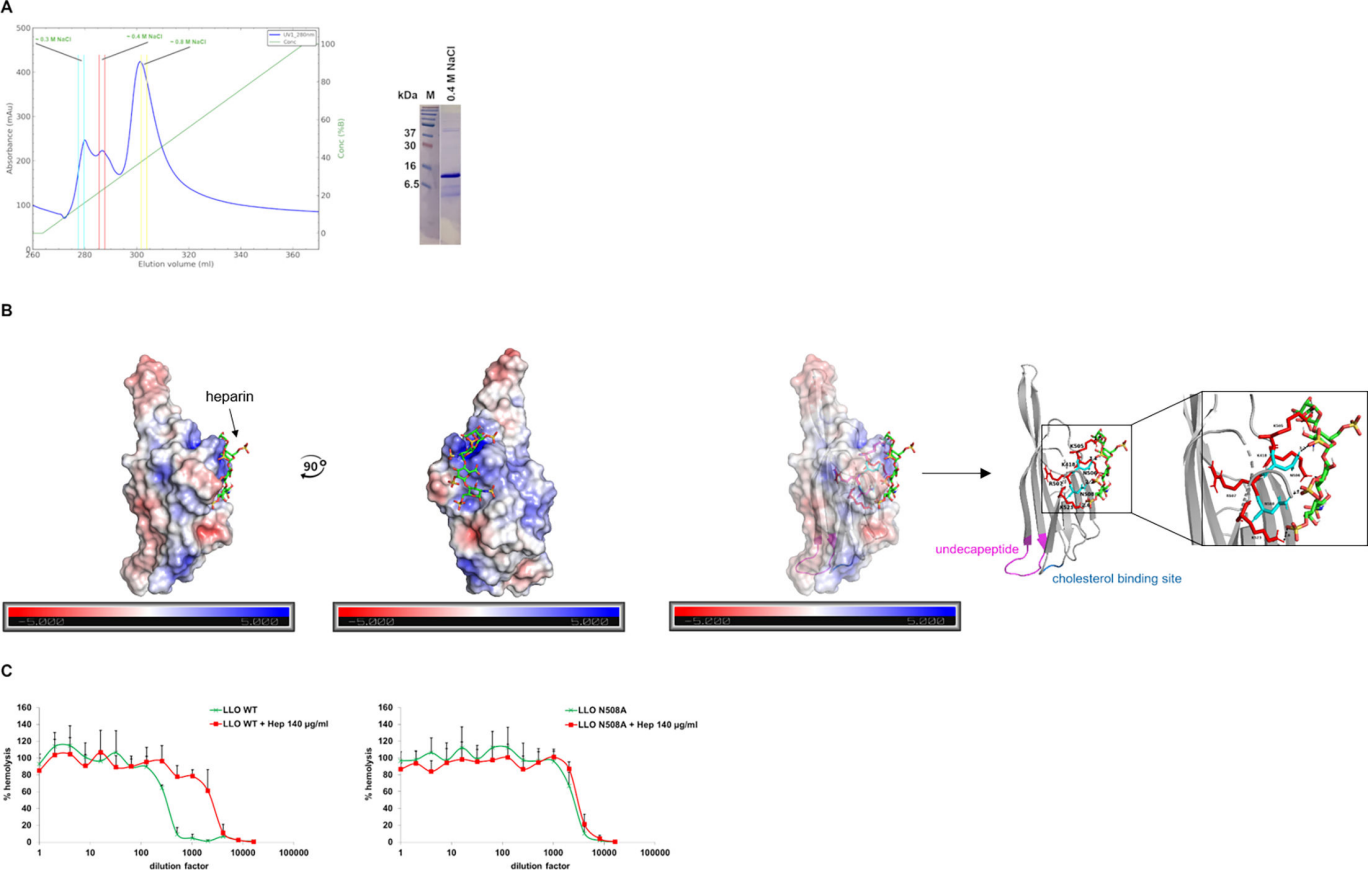
(**A**) (left): FPLC chromatogram output from HiTrap heparin affinity column of supernatant from *L. innocua* pIMK2_D4. Colored bars indicate selected elution fractions. Bound proteins were eluted with a linear gradient of elution buffer (0–2 M NaCl). Proteins eluted as three major peaks at salt concentrations of 0.3 M NaCl (blue), 0.4 M NaCl (red), and 0.8 M NaCl (yellow). Flow rate was at 4 mL/min, and elution fractions were collected every 50 s. mAU indicates absorption at 260 nm. (**A**) (right): Coomassie Brilliant Blue staining of protein isolation. Shown is the elution fraction at 0.4 M NaCl. The arrow indicates the protein band corresponding to D4. (**B**) Structural model of D4 highlighting the predicted heparin-binding site. Surface electrostatic potential is shown (red, negative; blue, positive; scale −5 to +5). Heparin (stick representation) is docked onto a positively charged surface patch. A 90° rotation illustrates the spatial orientation of the binding site. A schematic representation of the LLO structure (right) indicates the location of the undecapeptide region and the cholesterol-binding site relative to the heparin-binding pocket, with a zoomed view showing residues involved in heparin interaction. (**C**) Hemolytic activity assays of wild-type and mutant LLO in the presence or absence of heparin. Percent hemolysis is plotted against dilution factor for wild-type LLO (left) and the N508A mutant (right). Green curves represent toxin alone, while red curves indicate toxin incubated with heparin (140 μg/mL). Mean values ± SEM are plotted from three independent experiments.

As seen above, the addition of heparin and cholesterol has strong reciprocal effects on the hemolytic activity of LLO. Since the D4 domain is required for the recognition of cholesterol and required for toxin insertion into the target membrane, we considered that it also interacts with the C4 domain of LLO. To examine that D4 is indeed involved in heparin-binding, we fused the coding sequence for the D4-domain to the signal peptide of LLO in the integrative vector pIMK2 and overexpressed the gene using the constitutive promoter P_help_ in *Listeria innocua* (see Materials and Methods). Concentrated crude supernatants of *L. innocua* pIMK2_D4 grown overnight were loaded onto a sepharose-based heparin affinity column, eluted by a linear gradient of elution buffer, and the fractions were collected. Unlike LLO, D4 was eluted at lower salt concentrations (0.4 M NaCl) (Fig. 5A). The D4 domain was eluted as a single polypeptide of ~13 kDa. Its identity was confirmed by MALDI-TOF analysis.

To examine the potential binding sites within D4 for heparin, we used ClusPro to study its interaction with heparin (42). As shown in Fig. 5B, there is a prominent electropositive surface, which encompasses a significant portion of the region’s lateral and distal to the surface’s tryptophan-rich undecapeptide (UDP) and three distal loops (L1–L3), of which L1 contains the cholesterol recognition motif. While the loops are essential for membrane binding, the rest of the D4 domain projects away from the membrane surface and presents a large surface area for potential interactions with charged molecules such as heparin. As depicted in Fig. 5B, docking studies indicated that a region of charged residues exhibiting preferred interactions with heparin involved the amino acid residues K418, K505, K506, N508, and K523. Based on this prediction, we created an LLO variant by mutating the amino acid residue 508 from N to A. Asparagine residues are part of the XBBXBX motif for heparin binding sites, and their polar amide groups are required for hydrogen bridge bonds to the hydroxy- or sulfate-groups of heparin. As seen in Fig. 5C, although the purified LLON508A protein retained hemolytic activity, this activity was not enhanced by the addition of heparin. Thus, the N508 residue of LLO is essential for its interaction with heparin.

### Specificity of LLO and D4-domain for sulfated heparin oligosaccharides

**Fig 6:**
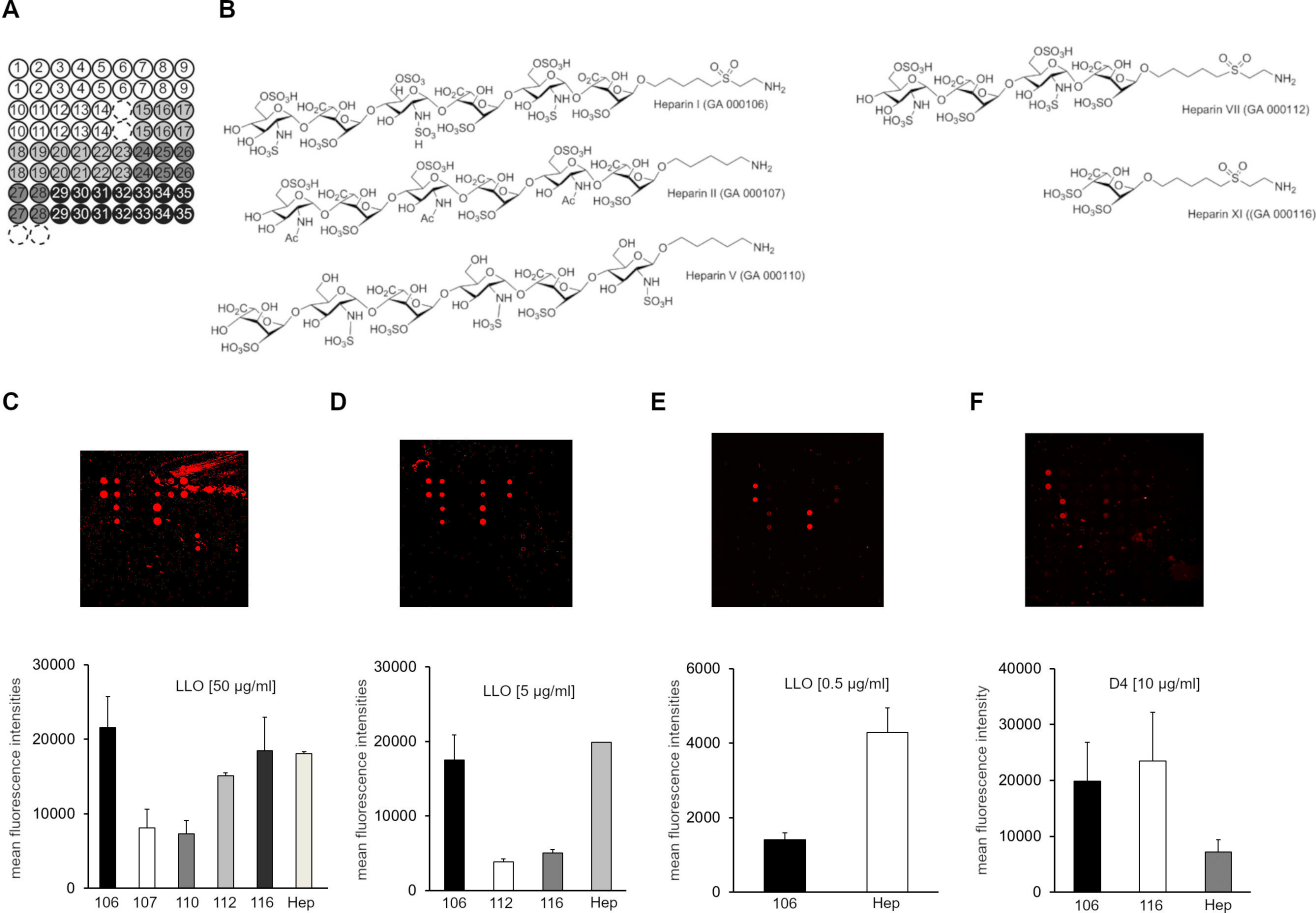
(**A**) Printing pattern of the glycan array. (**B**) Heparin structures that were identified as binders from the glycan arrays. (**C through E**) Representative field images of LLO interacting with the array at the indicated concentrations and the respective mean fluorescence intensities. Mean values ± SD are plotted from two independent experiments. (**F**) Representative field images of D4-domain (10 µg/mL) interacting with the array and the respective mean fluorescence intensities. Mean values ± SD are plotted from two independent experiments.

We used purified LLO and its D4-domain to probe arrays of sulfated heparin oligosaccharides and examined for optimal sulfation patterns and a minimal oligosaccharide structure. Based upon the major structural sequences seen with heparin, a series of di-, tetra-, and hexa-saccharides of the glucosamine-iduronic acid (GlcN-IdoA) repeating unit was synthesized. In addition, different sulfation patterns were introduced via position 2 of the iduronic acid units and positions 2 and 6 of the glucosamine units (Fig. 6A and B). Deaminated heparin obtained from natural sources with an average molecular weight of ~5 kDa was included as a control (Hep).

Arrays were probed with different concentrations of LLO. We detected concentration-dependent specific binding to the oligosaccharide structures (GA00106, GA00117, GA00110, GA00112, and GA00116) (Fig. 6C through E). These results suggest that the fully sulfated structures are involved in the interaction in LLO-glycan interactions. We confirmed the specificity of heparin and the longer GA00106 structure employing a competition assay where increasing concentrations of heparin are used to titrate initial binding of the toxin to either heparin itself or the GA00106 oligosaccharide (Fig. S6A and B). We also detected binding of the D4 domain to the same structures obtained with LLO (Fig. 6F). Competition assays performed as described above indicated the strong specificity of D4 binding to GA00106-, GA00116-, and Hep-sulfated oligosaccharides (Fig. S6C through E).

## DISCUSSION

In this study, we demonstrate that heparin is a novel enhancer of extracellular LLO activity. LLO-heparin complexes are highly active at physiological pH, and we use this information to develop a simple one-step isolation protocol for LLO from different *L. monocytogenes* serotypes that express varying levels of the toxin. The hemolytic activity of heparin-bound LLO is insensitive to inhibition by cholesterol and is not pH-dependent. Using synthetic heparin microarrays, we found that LLO binds only to fully sulfated repeating trisulfated disaccharide units and its substructures, a result that was replicated using a derivative comprising only its D4 domain. Molecular docking studies identified a potential heparin binding site within D4, and a mutant LLO derivative harboring a mutation therein still retains hemolytic activity, which, however, is not enhanced by the addition of heparin.

**Fig 7:**
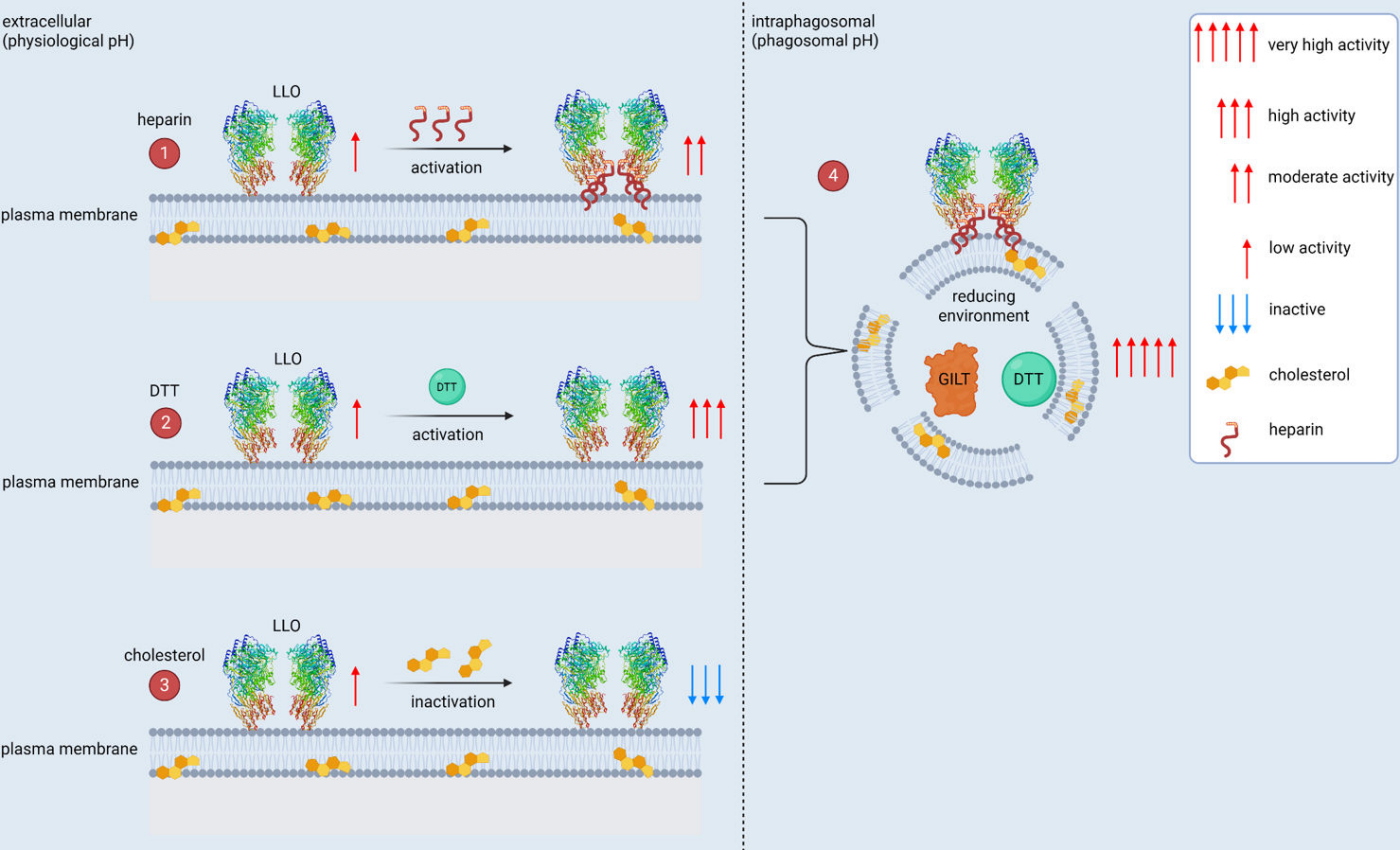
Summary of extracellular and intracellular modulation of LLO activity. Addition of heparin increases LLO activity from low activity to moderate activity (1). LLO treatment with DTT increases toxin activity from normal activity to high activity (2). Presence of free cholesterol inactivates LLO (3). The combined activity modulation by a reducing environment, together with heparin presence and an acidic pH, leads to a very high toxin activity (4) (Created in BioRender. Abu Mraheil, M. (2025) https://BioRender.com/rf2wnti).

LLO exhibits maximal pore-forming activity under the acidic conditions (~ pH 5.5), but it rapidly aggregates at pH values and temperatures of above 7 and 30°C, respectively. Toxin forms resulting from these conditions are incapable of interacting with lipid membranes and are non-hemolytic (27, 28). This pH-based regulation of LLO activity is crucial for the pathogenic potential of *L. monocytogenes* and is underlined by studies with a mutant expressing a variant toxin form (L461T) that demonstrated high hemolytic activity at neutral pH but exhibited a 100-fold reduction in virulence due to its excessive cytotoxicity (43). LLO also has important roles in extracellular sites as it has been shown that *L. monocytogenes* replicates in the gut and gall bladder and that the toxin is required for its dissemination from these sites to establish infection in systemic organs (44–46). LLO could enhance its extracellular activity conditionally by using regulatory principles similar to those used in modulating its intracellular activity. Thus, host-specific factors that facilitate binding and enhance its activity locally would contribute further to the versatile properties of the toxin. Our data suggest that engaging heparin and its derivatives represents a mechanism for transient, localized, and reversible enhancement of LLO activity (Figure 7).

Our data originated from an assessment of the secreted protein fraction of *L. monocytogenes* that identified several water-soluble protein species with heparin-binding properties. They included three bacterial proteins with autolytic (Fig. 2C) viz. the virulence-associated Iap protein (40, 47) and Spl (48), a hitherto uncharacterized protein designated *L. monocytogenes* 0129 (Lmo0129), as well as LLO as heparin-binding proteins. We focused our attention on LLO, as it was a secreted protein with the highest affinity for heparin and represents a major virulence factor of *L. monocytogenes*. The observation that LLO binds heparin was a general property, regardless of the isolate studied, and enabled us to develop a simple one-step isolation protocol for this toxin. The ability to purify LLO from any isolate is highly relevant, as clinical isolates of *L. monocytogenes* often exhibit weak and variable hemolytic phenotypes (26, 49), so that in-depth qualitative studies of LLO produced by these isolates may be carried out.

We also found that the related CDC pneumolysin (PLY) could be affinity-purified using a heparin sepharose (Fig. S4A). Heparin-dependent activation of the hemolytic activity PLY was much lower at 1.6-fold than that of LLO (~ 10-fold) and could be achieved only with high concentrations of heparin (Fig. S4B). PLY also binds the Sialyl LewisX (sLeX) antigen on human red blood cells, and the sLeX glycosylated CD11b component of Mac-1 (Macrophage-1 antigen) has been identified as a cellular receptor for PLY on human phagocytes (50). We note that there is strong evidence showing that ligand binding to CD11b is blocked by heparin, suggesting that there are overlapping binding capacities for both sLeX and heparin for this receptor (25).

We showed that the PEST region and its conserved cysteine residue LLO are not required for heparin-enhancing hemolytic activity of LLO. Activation of LLO activity by heparin and DTT was synergistic. It increased by around 10-fold in the presence of heparin alone or 16-fold with only DTT and 186-fold in the presence of both reagents (Fig. S5A). As both reducing conditions and increasing acidification are properties associated with maturating phagosomes, these observations provide us with a powerful tool to assess and manipulate these activities experimentally.

Heparin binds to the C-terminal D4 domain of LLO, an observation that is consistent with previous observations found in studies with other glycans (50). Structures within D4 include four loops that are essential for initiating membrane contact during the pore-forming process (Fig. 5B). Three of these are short hydrophobic loops, while the fourth is a tryptophan-rich loop that harbors the undecapeptide sequence at its tip. The highly conserved (among CDCs) Thr515-Leu516 pair required for cholesterol binding is in L1, as is the Trp512 residue that is required for membrane binding. These membrane-interacting loops (L1, L2, L3, and the UDP) are highly conserved among the CDCs; however, the unique electrostatic profile of the remainder of its D4 domain of LLO sets it apart from other members of the family of cholesterol-dependent cytolysins (2). Molecular docking studies indicated preferred interactions with an electropositive surface that is diametrically distal to the UDP sequence and comprises a lateral region spanning the amino acid residues between K418 and K523 (Fig. 5B). Heparin binding would be predicted to align parallel to the membrane plane and bridge both the UDP and membrane insertion loops. The cationic residues near this interface would provide a stabilizing, neutralizing charge that potentially protects the toxin from premature aggregation and denaturation at physiological pH.

Glycan arrays were used to identify specific, structurally defined glycan fragments recognized by LLO. The synthetic heparin hexasaccharide (Heparin I, GA000106) exhibited the highest relative fluorescence units (RFU) during incubation with LLO, with a significant loss observed with its 6-O-desulfated Heparin II (GA000107) derivative (Fig. 6C). As previously reported, while Heparin II still possesses the N-sulfate and 2-O-sulfate groups and retains significant electrostatic potential, the absence of the 6-O sulfate group greatly decreases the strength of this interaction (51). This suggests that the presence of hydrogen-bond donors such as asparagines, which interact with oxygen atoms of the 6-O sulfate group, strongly drives this interaction and confers spatial orientation to it (52). These results were replicated using the recombinant D4-domain derivative of LLO (Fig. 6F). As the polar amide group of the asparagine side chain is known to form hydrogen bonds with the hydroxy or sulfate groups of heparin, we found that the variant LLO N508A impacted its ability to utilize heparin as an enhancer of hemolytic activity (Fig. 5C).

The C-terminal D4 domain of LLO is also the binding site of the family of C-series of gangliosides, and studies with a free GT2 glycan showed that it blocked LLO-mediated hemolytic activity against human red blood cells (RBCs) (50). Unlike interactions described for exogenous carbohydrate glycan receptors that inhibit, heparin clearly increases hemolytic activity. Further studies are required to determine their respective binding sites and examine how these binding interactions lead to opposing activities or whether these simply reflect effects of concentration dependence.

A role for host cell heparan sulfates (HS) in listerial pathogenesis was recognized over 25 years ago. These early studies reported that two surface-bound proteins, ActA and InlB, interact with mammalian cell surface HS to enable host cell entry through exploitation of the host cell endocytic machinery (35, 36, 53, 54). In addition, LLO has also been reported to promote cellular invasion by *L. monocytogenes* in epithelial cells (8, 55). Recently, a major family of the HS-containing proteoglycan Syndecan-1 (Sdc-1) was found to promote listeriosis (38). However, *L. monocytogenes* does not use Sdc-1 for either its attachment or invasion but instead promotes the shedding of the Sdc-1 ectodomains, preventing its clearance by intravascular aggregated neutrophil extracellular traps (NETs) in the livers of infected mice.

Heparan sulfate proteoglycans (HSPGs), such as syndecans, glypicans, and perlecans, are ubiquitously expressed on cell surfaces and the extracellular matrix (ECM) and harbor the fully sulfated repeating trisulfate disaccharide motif described here on specific domains within the glycan chain, where they bind specifically and non-covalently to proteins (51). HSPGs are generally considered to function as co-receptors for heparin-binding microbial pathogens, where they increase the concentration of these bacteria at the host surface, facilitating interactions with their respective signaling receptors (56). As heparin appears to be a common interacting partner for several proteins on the surface of *L. monocytogenes*, a detailed analysis will be necessary to create mutants that enable the dissection of a role for HSPGs during infection. Since heparin is produced by connective tissue-type mast cells and bipotential glial progenitor cells and found in the extracellular matrix and basement membranes, it is likely that cell-specific interactions of *L. monocytogenes* with different cell types could contribute to tropism and pathogenesis seen with this bacterium.

## MATERIALS AND METHODS

### Bacterial strains and growth conditions

Bacterial strains used in this work are presented in Table 1.

All strains were grown either in brain heart infusion (BHI) medium or in minimal medium (57).

**TABLE 1:** Bacterial strains used in this study*^a^*

Strain	Genotype	Serotype	Ref
*Lm* EGD-e	Wildtype	1/2a	(58)
*Lm* SLCC2755	Wildtype	1/2c	(59)
*Lm* SLCC2372	Wildtype	1/2c	(59)
*Lm* L99	Wildtype	4a	(60)
*Lm* EGD-e Δ*iap*	Iap deletion mutant	1/2a	(47)
*Lm EGD-e Δhly*	Hly deletion mutant	1/2a	(61)
*Lm* EGD-e LLO C484S	Substitution C → S	1/2a	(41)
*Linn* Δ*lgt pIMK2_PLY*	His-tag PLY expressing	6a	(17)
*Linn* Δ*lgt pIMK2*_*D4*	His-tag D4 expressing	6a	This work
*Linn* Δ*lgt pERL3_N508A*)	Substitution N → A	6a	This work

^*a*^ Lm = *L. monocytogenes*; Linn = *L. innocua*.

### Construction of *L. innocua* CLIP11266 Δ*lgt* pIMK2_PLY and *L. innocua* CLIP11266 Δ*lgt* pIMK2_D4

For isolation of PLY, *ply* was amplified by PCR using the primers PlyBamHI_for (5′-GCAAAGGGATCCGCAAATAAAGCAGTAAATGACTTTATAC-3′) and PlyXhoI_rev (5′-CATTCTCTCGAGCTAGTCATTTTCTACCTTATCCTCTACC-3′) from *S. pneumoniae* D39 (62). The PCR product was digested with *BamH*I and *Xho*I restriction enzymes and ligated into a *BamH*I/*Xho*I-digested pIMK2 vector (63). To create a N-terminal His-tagged PLY, His-tag coding sequence was inserted within the *BamH*I restriction site by using the phosphorylated and annealed primers HisPly_for (5′-GATCCCATCATCATCATCATCACAGCAGCGGCCTGGTGCCGCGCGGCAGC-3′) and HisPly_rev (5′-GATCCGCTGCCGCGCGGCACCAGGCCGCTGCTGTGATGATGATGATGATGG-3′). LLO signal peptide for protein secretion was amplified from *L. monocytogenes* EGD-e using the primers *SphIIPagI_*for (5′-TGAAACTCATGAAAAAAATAATGCTAGTTTTTATTACACTTATATTAG −3’) and *SphIPagI*_rev (5′-TGAATTGGATCCACCGCTGCCAGATGCATCCTTTGCTTCAGTTTGTTGC-3′) and ligated into the *Nco*I/*BamH*I restriction site of pIMK2. The final construct expresses PLY under the control of the constitutive promoter P_help_ and includes a signal peptide from LLO for protein secretion (64). *E. coli* DH10β was transformed with the constructed plasmids, and the sequence was verified by Sanger sequencing. The final construct was integrated into the chromosome of *L. innocua* ∆*lgt* (65).

For the isolation of the D4-domain, the LLO gene sequence from aa 418–529 was amplified by PCR using the primers D4_LLO_Xho_f (5′-GCGCGCCTCGAGACAGATGGAAAAATT-3′) and D4_LLO_Kpn_r (5′-GCGCGCGGTACCTTATTCGATTGGATT-3′) from *L. monocytogenes* EGD-e (58). The PCR product was digested with *Xho*I and *Kpn*I restriction enzymes and ligated into a *XhoI*/*Kpn*I-digested pIMK2 vector (63). To create an N-terminal His-tagged domain, D4 His-tag coding sequence was inserted within *BamH*I and *Xho*I restriction sites by using the phosphorylated and annealed primers HisBam_for (5′-AGCGGTGGATCCCATCATCATCATCATCACAGC-3′) and HisXho_rev (5′-TTTAAACTCGAGGCTGCCGCGCGGCACCAGGC-3′). The LLO signal peptide for protein secretion was amplified from *L. monocytogenes* EGD-e using the primers *SphIIPagII*_for (5′-TGAAACTCATGAAAAAAATAATGCTAGTTTTTATTACACTTATATTAG −3’) and *SphIIPagI*_rev (5′-TGAATTGGATCCACCGCTGCCAGATGCATCCTTTGCTTCAGTTTGTTGC-3′) and ligated into the *BamH*I/*Xho*I restriction sites of pIMK2. The final construct expresses the D4-domain under the control of the constitutive promoter P_help_ and includes a signal peptide from LLO for protein secretion between P_help_ and *ply. E. coli* DH10β was transformed with the constructed plasmids, and the sequence was verified by Sanger sequencing. The final construct was integrated into the chromosome of *L. innocua* ∆*lgt* (61).

### Construction of *L. innocua* CLIP11266 Δ*lgt*_LLO N508A

For site-directed mutagenesis of LLO, the plasmid pERL-3 501 LLO wt (66) was used as a template. The N508A substitution was introduced using the primers LLO N508A_for (5′-GAAAAATAGAGCTATCTCCATCTGGGGC-3′) and LLO N508A_rev (5′-ACAAGTGGTAAGTTCCGG-3′). Mutagenesis was performed using the Q5 Site-Directed Mutagenesis Kit (New England Biolabs) according to the manufacturer’s instructions. Following PCR amplification, the reaction mixture was treated with kinase, ligase, and DpnI to circularize the plasmid and remove the template DNA. The resulting construct was transformed into chemically competent *E. coli* cells and plated on a selective medium. Plasmid DNA from positive clones was isolated, and the presence of the N508A mutation was confirmed by Sanger sequencing. Protein isolation was performed as described previously (67).

### Concentration of crude bacterial supernatant

Bacteria were grown overnight at 37°C in minimal medium (68). Stationary phase bacteria were pelleted by centrifugation (6,000 rpm, 10 min, 4°C), and the crude supernatant was loaded onto centrifugal filter units (Amicon Ultra - 15, Merck Millipore), with a cutoff of 10 kDa. The supernatant was concentrated by several centrifugation steps (4,000 × *g*, 15 min, 4°C) before the medium was subsequently exchanged to binding buffer (10 mM sodium phosphate, pH 7.0). Concentrated supernatants were either directly used as described below or flash frozen and stored at −80°C in liquid nitrogen.

### Bulk purification of LLO

For affinity purification in batch format, heparin Sepharose beads (Heparin Sepharose 6 Fast Flow, GE Healthcare) were washed three times with binding buffer (10 mM sodium phosphate, pH 7.0) by centrifugation at 1,000 × *g* for 2 min at room temperature; 1 ml of concentrated supernatant was added to equilibrated heparin beads and incubated for 1 h on a rotating device at room temperature. Incubated beads were centrifuged (1,000 × *g*, 2 min, room temperature) and washed three times with binding buffer. For the elution of bound proteins, an elution buffer with increasing salt concentrations was used (10 mM sodium phosphate, 0.4–2 M NaCl, pH 7.0). In between the elution steps, beads were washed three times with binding buffer. Washing and elution fractions were collected and used for subsequent SDS-PAGE.

### Ion-exchange chromatography for protein purification

Isolation of LLO wt and LLO_C484S_ mutant by ion exchange chromatography was performed as described previously (17, 67).

### Heparin sepharose-based protein purification

For heparin sepharose-based protein purification, an overnight bacterial culture of *L. monocytogenes* wt (LLO isolation), *L. innocua CLIP11266* Δ*lgt* pIMK2*_*PLY (PLY isolation), *L. innocua CLIP11266* Δ*lgt* pIMK2*_*D4 (D4 isolation), and *L. innocua CLIP11266* Δ*lgt pERL3_*N508A (LLO N508A isolation) in BHI was used to inoculate (0.5%) 2 L of minimal medium (69) and cultured at 30°C without shaking for 72 h. The bacterial culture was centrifuged at 9,000 rpm at 4°C for 30 min. The supernatant was collected, sterile-filtered (RapidFiltermax, TPP), and concentrated to approximately 30 mL using cross-flow filtration using MiniKros Sampler Hollow Fiber Filters (#S02-E010-05-N, Repligen). During filtration, the buffer was exchanged to binding buffer (10 mM sodium phosphate, pH 7.0).

The concentrate was loaded onto a 5 mL HiTrap Heparin HP affinity column (#17040601, GE Healthcare) using fast protein liquid chromatography (ÄKTA explorer and UNICORN control system; GE Healthcare) at ambient temperatures and washed with seven column volumes of binding buffer (10 mM sodium phosphate, pH 7.0) to eliminate unbound and non-specific-binding proteins. Proteins that bound to the heparin sepharose resin were eluted by a linear gradient of elution buffer (10 mM sodium phosphate, 0-100%; 0–2 M NaCl, pH 7.0). The flow rate was 4 mL/min. Chromatograms were created with PyCORN-Web (http://pycornweb.appspot.com/). Fractions were subsequently dialyzed against PBS, flash frozen, and stored in liquid nitrogen at −80°C.

### Determination of protein concentration

Estimation of protein concentration was performed using the Bradford assay (68). In short, protein-containing suspensions were diluted appropriately in elution buffer. Ten microliters of each sample were pipetted into a flat 96-well microplate, and 200 µL of protein assay dye reagent working solution (Protein Assay Dye Reagent Concentrate, Biorad #500-0006, 1:5 with H_2_O) was added. The plate was mixed shortly on a plate shaker and incubated for 10 min at room temperature (RT), and the absorption at a wavelength of 595 nm (OD_595 nm_) was measured by microplate reader. The amount of protein within the sample was estimated by comparison to a bovine serum albumin standard curve.

### Western blot analysis

Proteins were loaded onto SDS gels, subjected to SDS-PAGE, and transferred to PVDF membranes. The membranes were subsequently incubated at room temperature (RT) for 1 h in blocking buffer (5% non-fat dry milk in TBS with 0.1% Tween-20) and incubated with in-house anti-LLO primary antibody (M275) (70) or in-house anti-Iap primary antibody (Fup 60) at 4°C overnight. Western blots were incubated the next day with HRP-coupled secondary antibody at RT for 1 h. Data acquisition occurred by the addition of the ECL substrate and subsequent exposure to X-ray films.

### Hemolysis assay

Hemolytic activity of LLO or PLY was determined by lysis of sheep erythrocytes. Whole blood from sheep was centrifuged at 3,800 rpm for 3 min and 4°C to pellet erythrocytes and washed three times with hemolysis buffer (150 mM NaCl, 50 mM sodium phosphate, pH 6.6) to remove traces of heparin and serum. For pH experiments, pH was adjusted accordingly. Washed erythrocytes were then diluted in hemolysis buffer (supplemented with 5 mM dithiothreitol where needed) to make an erythrocyte suspension (1%). Elution fractions or pooled toxin-containing fractions were serially diluted (1:2) in a V-shaped 96-well microplate in a total volume of 50 μL. Heparin (Santa Cruz Biotechnology, #sc-203075), DNA (Sigma-Aldrich, #D1501), RNA (Roche, #10109223001), tRNA (Sigma-Aldrich, #R8759), cholesterol (Merck, #3670), and phosphocholine (Sigma-Aldrich, #C7017) were added where stated. The hemolysis assay was started by adding 50 μL of the erythrocyte suspension to the microplate. The test was incubated for 1 h at 37°C. Water was used as a positive control. Data were collected by determining the optical density (OD) at a wavelength of 405 nm (OD_405 nm_) using a microplate reader (Multiskan FC, Thermo Scientific). Hemolysis was described as relative hemolysis to full hemolysis caused by H_2_O.

### Mass spectrometry

Protein solutions were digested with trypsin and then desalted and concentrated. MALDI-TOF-MS was performed on an Ultraflex TOF/TOF mass spectrometer (Bruker Daltonics, Bremen, Germany) equipped with a nitrogen laser and a LIFT-MS/MS facility. The instrument was operated in the positive-ion reflectron mode using 2.5-dihydroxybenzoic acid and methylenediphosphonic acid as matrix. Sum spectra consisting of 200–400 single spectra were acquired. For data processing and instrument control, the Compass 1.4 software package, consisting of FlexControl 4.4, FlexAnalysis 3.4, Sequence Editor, and BioTools 3.2, was used. External calibration was performed with a peptide standard (Bruker Daltonics) (71).

### Glycan array

The heparin glycan array was prepared as described previously (52, 72). Glycans containing a reactive aminolinker were dissolved (250 µM) in sodium phosphate (50 mM, pH 8.5). Solutions were printed onto CodeLink N-hydroxy-succinimide-activated glass slides (Surmodics, Eden Prairie, MN) by using an S3 piezoelectric non-contact microarray printer (Scienion, Berlin, Germany) equipped with a type-4-coated nozzle. Each slide was printed with 64 identical fields. Slides were incubated overnight in a humidity-saturated chamber and subsequently quenched by incubation with ethanolamine (100 mM) in sodium phosphate (50 mM, pH 9.0) for 1 h at RT. Slides were washed twice with water, dried by centrifugation (300 × *g*; Eppendorf CombiSlide system), and stored at 4°C. Slides were blocked for 30 min with PBS containing BSA (1%), washed twice with PBS, and dried by centrifugation. A 64-well incubation gasket (Flexwell Incubation Chamber, Grace Bio-Labs) was attached to the slide. Diluted purified LLO (50 µg/mL, 5 µg/mL, and 0.5 µg/mL) or D4-domain (10 µg/mL) was applied (20 µL per well) to the slide. For competition assays, toxins were preincubated with indicated concentrations of heparin for 30 min at RT (heparin sodium salt, Carl Roth). After incubation in a humidified chamber at RT for 1 h in the dark, wells were washed three times with TBS containing Tween-20 (0.1%, pH 7.4). Primary anti-LLO antibody (Abcam, 1:200) was applied to the slide and incubated in a humidified chamber for 1 h in the dark at RT. After a washing step (three times) with TBS-T, the secondary anti-rabbit antibody was applied (Alexa Fluor 647, 1:400) and incubated as described before. The entire slides were subsequently washed twice with TBS-T, once with TBS, rinsed with H_2_O, and the multiwall gasket was removed. After drying the slides by centrifugation, the slides were scanned on a GenePix 3400 A microarray scanner (Molecular Devices), and the intensities of the spots (diameter 160 µm) were evaluated with GenePix 7.2 software (Molecular Devices), with local background subtracted from mean intensity.

### Statistical analysis

Statistical analysis of experiments was performed with SigmaPlot 11 (Systat Software). A *P* value of *≤ 0.05, **≤ 0.01, and ***≤ 0.001 were considered statistically significant. The number of individual experiments is indicated in the Results section.

## SUPPLEMENTAL MATERIAL

Supplemental figure legends jb.00526-25-s0001.docx Legends for Figures S1 to S6.

Figure S1 jb.00526-25-s0006.tif Sheep erythrocytes were incubated with pooled LLO-containing elution fractions (4.25 µg/mL) at 37°C for 1 h.

Figure S2 jb.00526-25-s0003.tiff FPLC chromatogram output from HiTrap heparin affinity column of supernatant from *L. monocytogenes* SLCC2755 (A), L99 (B), SLCC2372 (C).

Figure S3 jb.00526-25-s0004.tif Sheep erythrocytes were incubated with elution fraction (2 µg/mL) with highest hemolytic activity from *L. monocytogenes* EGD-e.

Figure S4 jb.00526-25-s0005.tif FPLC chromatogram output from HiTrap heparin affinity column of supernatant from *L. innocua* pIMK2_ply. Arrow indicates protein band corresponding to PLY in Coomassie Brilliant Blue stained SDS-PAGE loaded with indicated elution fractions.

Figure S5 jb.00526-25-s0002.tif LLO wildtype (wt) (1.25 μg/mL) and LLOC484S (20 μg/mL) were treated simultaneously with heparin (1 µg/mL) and incubated with sheep erythrocytes at 37°C for 1 h under nonreducing (− DTT) or reducing (+ DTT) conditions.

Figure S6 jb.00526-25-s0007.tif Mean fluorescence intensities of the glycan array with printing of Heparin I (GA 000106).

Table S1 jb.00526-25-s0008.tiff Isolates of *L. monocytogenes* tested.

ASM does not own the copyrights to Supplemental Material that may be linked to, or accessed through, an article. The authors have granted ASM a non-exclusive, world-wide license to publish the Supplemental Material files. Please contact the corresponding author directly for reuse.
