# Supplementary material for: Heparin-binding enhances extracellular listeriolysin O activity, overcoming cholesterol inhibition and pH dependence
Source: J Bacteriol. 2026 Jun 12;208(7):e00526-25. doi: 10.1128/jb.00526-25 (PMC13393555; doi:10.1128/jb.00526-25)
Supplement: Supplemental figure legends — Legends for Figures S1 to S6. [file jb.00526-25-s0001.docx]

# Supplemental information

Table S1: Isolates of *L. monocytogenes* tested. The hemolytic units (HU) are calculated from the elution fraction that showed highest hemolytic activity. The hemolytic units are defined as the amount of toxin at which 50% of the red blood cells (RBC) had been lysed by H_2_O (pos. CTRL).

Figure S1: Sheep erythrocytes were incubated with pooled LLO-containing elution fractions (4.25 µg/mL) at 37°C for 1 h. After centrifugation, the optical density (OD) at a wavelength of 405 nm (OD_405 nm_) was measured.

Figure S2: A: FPLC chromatogram output from HiTrap heparin affinity column of supernatant from *L. monocytogenes* SLCC2755 (A), L99 (B), SLCC2372 (C). Colored bars indicate selected elution fractions. Bound proteins were eluted with a linear gradient of elution buffer (0-2 M NaCl). Proteins eluted as three major peaks at salt concentrations of 0.6 M NaCl (blue), 0.8 M NaCl (red) and 1.0 M NaCl (yellow). Flow rate was at 4 mL/min and elution fractions were collected every 50 s. mAU indicates absorption at 260 nm.

Figure S3: A-D: Sheep erythrocytes were incubated with elution fraction (2 µg/ml) with highest hemolytic activity from *L. monocytogenes* EGD-e (A), *L. monocytogenes* SLCC2755 (B), *L. monocytogenes* L99 (C) or *L. monocytogenes* SLCC2372 (D) in the presence or absence of heparin (1 µg/mL) at 37°C for 1 h. After centrifugation, the optical density (OD) at a wavelength of 405 nm (OD_405 nm_) was measured. Hemolytic activity is increased by 10-fold after heparin addition (red squares) as compared to the untreated control (green crosses). Mean values ± SEM are plotted from 3 independent experiments. Addition of other highly negative charged macromolecules (DNA, RNA, tRNA) have no influence on hemolytic activity (E). Addition of heparin increases also hemolytic activity from LLO isolated by ion exchange chromatography 10-fold (F). Mean values ± SEM are plotted from 3 independent experiments (* P ≤ .05; Student's t-test).

Figure S4: A: FPLC chromatogram output from HiTrap heparin affinity column of supernatant from *L. innocua* pIMK2_ply*.* Arrow indicates protein band corresponding to PLY in Coomassie Brillant blue stained SDS-PAGE loaded with indicated elution fractions. B: Sheep erythrocytes were incubated with pooled PLY-containing elution fractions (300 µg/mL) in the presence of indicated heparin concentrations at 37°C for 1 h. After centrifugation, the optical density (OD) at a wavelength of 405 nm (OD_405 nm_) was measured. B: Sheep erythrocytes were incubated with pooled PLY-containing elution fractions (300 µg/mL) in the presence of indicated heparin concentrations at 37°C for 1 h. After centrifugation, the optical density (OD) at a wavelength of 405 nm (OD_405 nm_) was measured.

Figure S5: A: LLO wildtype (wt) (1.25 μg/mL) and LLO_C484S_ (20 μg/mL) were treated simultaneously with heparin (1 µg/mL) and incubated with sheep erythrocytes at 37°C for 1 h under nonreducing (− DTT) or reducing (+ DTT) conditions. After centrifugation, the optical density (OD) at a wavelength of 405 nm (OD_405 nm_) was measured. The presence of DTT increased hemolytic activity of LLO wt ~ 16-fold, but had no effect on hemolytic activity of LLO_C484S_. The presence of heparin increased hemolytic activity of LLO_wt_ and LLO_C484S_ mutant ~ 10-12-fold. Co-treatment with DTT and heparin increased hemolytic activity of LLO wt 186 times and 8-fold in the LLO_C484S_ mutant. Mean values ± SEM are plotted from three independent experiments (* P ≤ .05; ** P ≤ .01; NS = not significant; Kruskal-Wallis test and Student-Newman-Keuls posthoc test). B: LLO *ΔPEST* (2.25μg/ml) was treated simultaneously with heparin (hep) (1 µg/mL or 100 µg/mL) and incubated with sheep erythrocytes at 37°C for 1 h. After centrifugation, the optical density (OD) at a wavelength of 405 nm (OD_405 nm_) was measured. The presence of heparin increased hemolytic activity of LLO *ΔPEST* as compared to the untreated control.

Figure S6: Mean fluorescence intensities of the glycan array with printing of Heparin I (GA 000106) (A) or deaminated heparin obtained from natural sources (Hep) (B) after preincubation of LLO with heparin at indicated concentrations (competition assay). Mean values ± SD are plotted from two independent experiments. C-E: Mean fluorescence intensities of the glycan array with printing of Heparin I (GA 000106), Heparin XI (GA 000116) or deaminated heparin obtained from natural sources (Hep) after preincubation of D4-domain with heparin at indicated concentrations (competition assay). Mean values ± SD are plotted from two independent experiments.
