# Supplementary figures and images for: Heparin-binding enhances extracellular listeriolysin O activity, overcoming cholesterol inhibition and pH dependence
Source: J Bacteriol. 2026 Jun 12;208(7):e00526-25. doi: 10.1128/jb.00526-25 (PMC13393555; doi:10.1128/jb.00526-25)

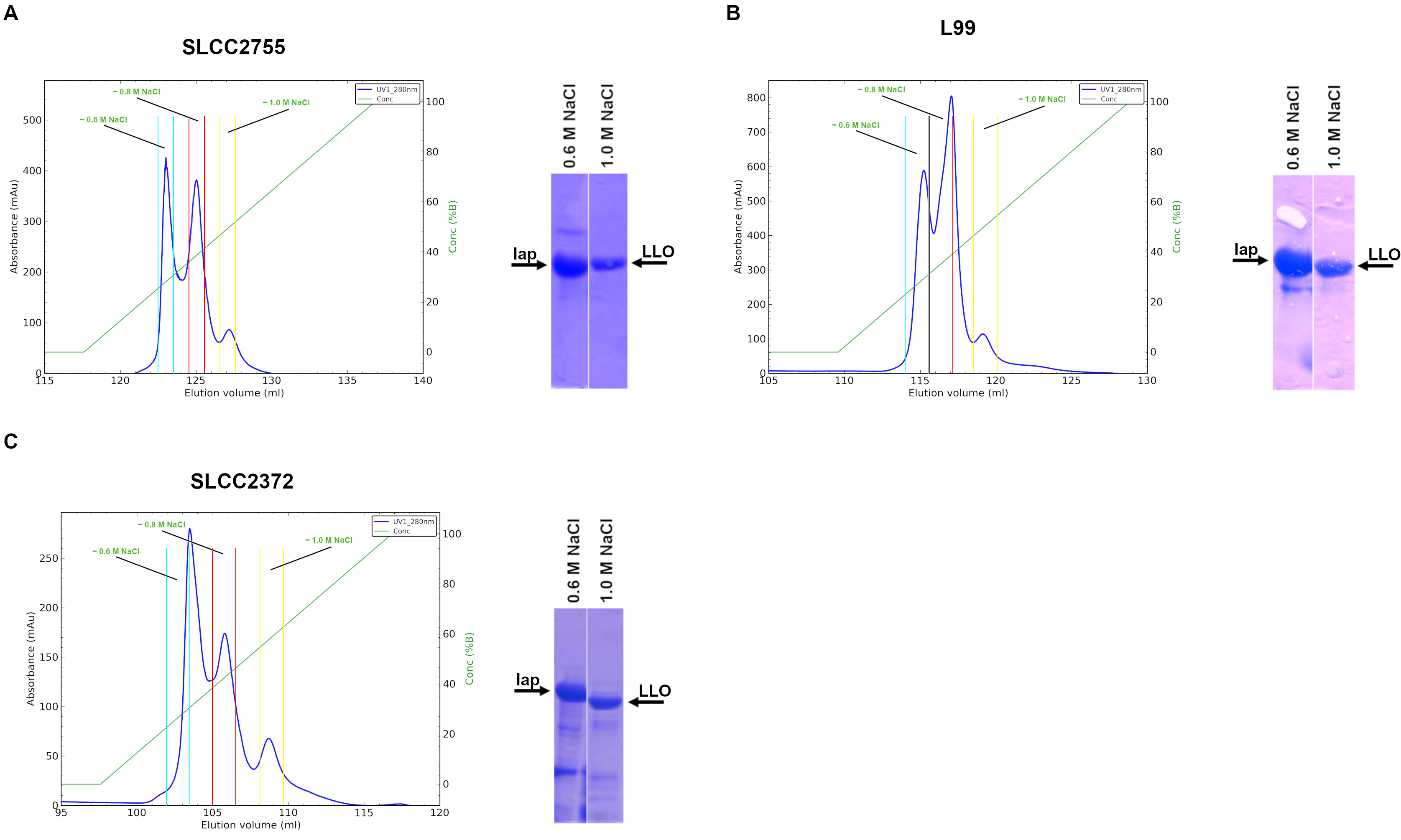

Supplement: Figure S1 — Sheep erythrocytes were incubated with pooled LLO-containing elution fractions (4.25 µg/mL) at 37°C for 1 h. [file jb.00526-25-s0006.tif]

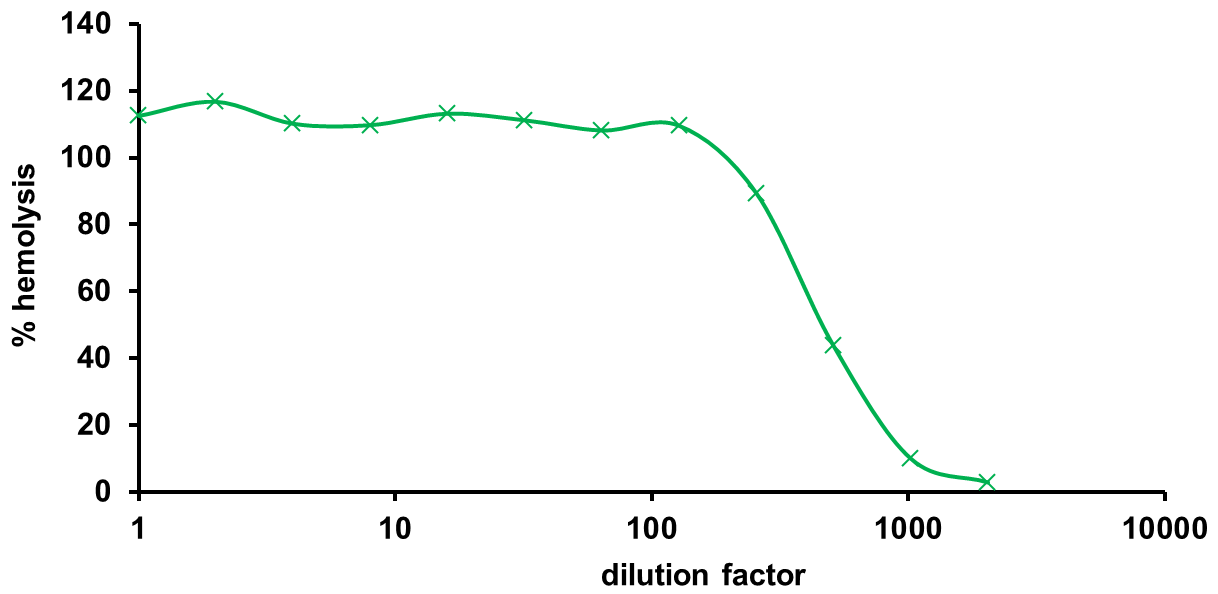

Supplement: Figure S2 — FPLC chromatogram output from HiTrap heparin affinity column of supernatant from L. monocytogenes SLCC2755 (A), L99 (B), SLCC2372 (C). [file jb.00526-25-s0003.tiff]

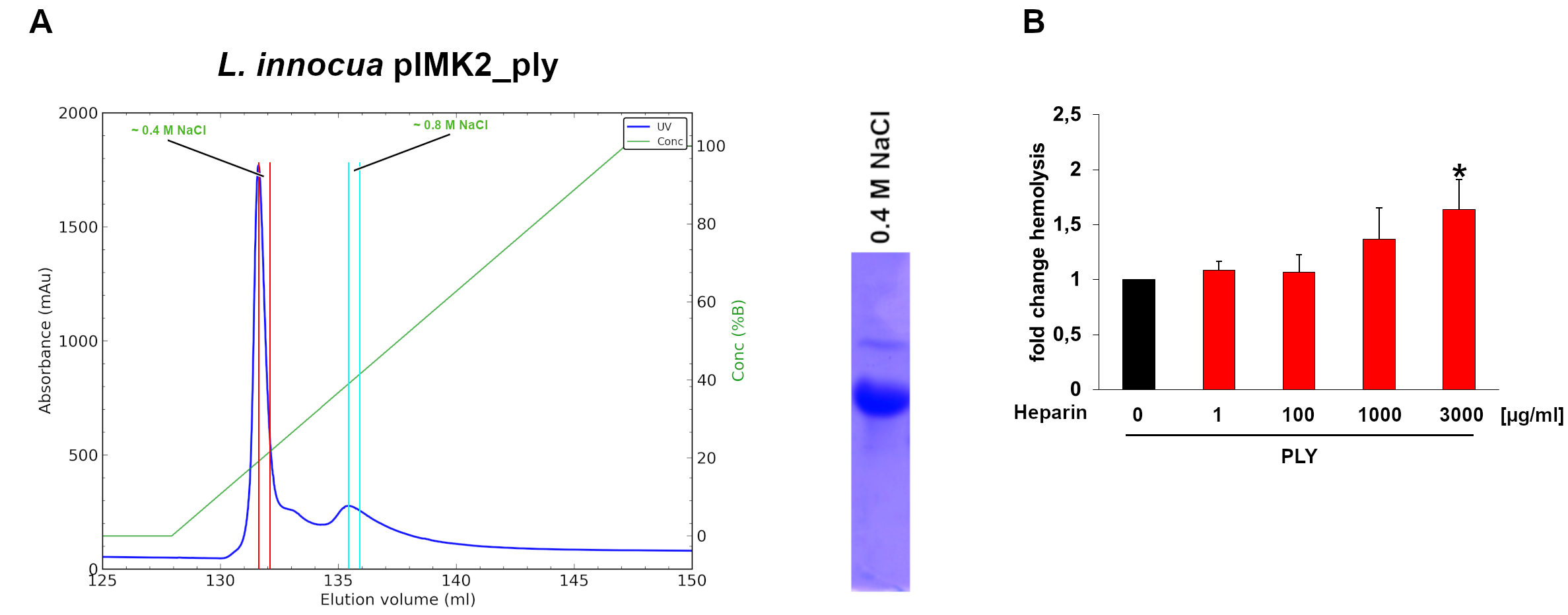

Supplement: Figure S3 — Sheep erythrocytes were incubated with elution fraction (2 µg/mL) with highest hemolytic activity from L. monocytogenes EGD-e. [file jb.00526-25-s0004.tif]

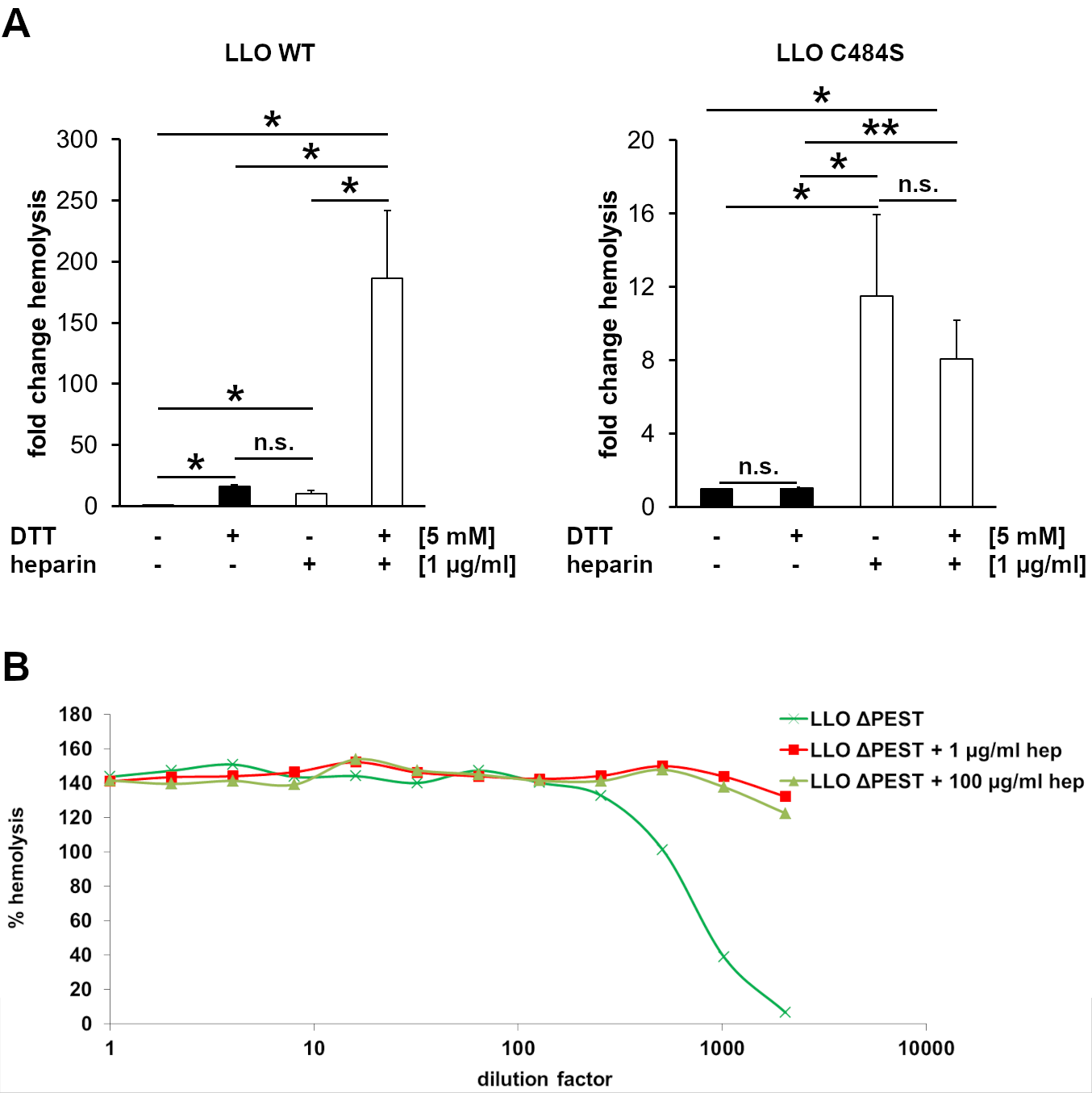

Supplement: Figure S4 — FPLC chromatogram output from HiTrap heparin affinity column of supernatant from L. innocua pIMK2_ply. Arrow indicates protein band corresponding to PLY in Coomassie Brilliant Blue stained SDS-PAGE loaded with indicated elution fractions. [file jb.00526-25-s0005.tif]

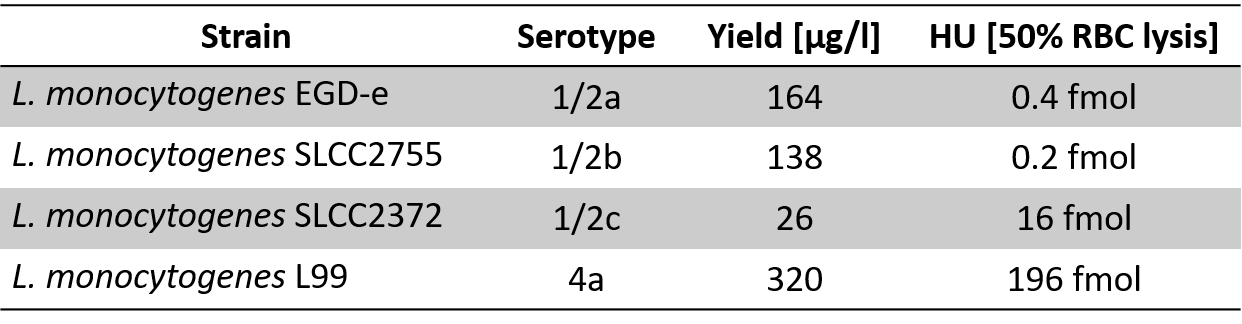

Supplement: Figure S6 — Mean fluorescence intensities of the glycan array with printing of Heparin I (GA 000106). [file jb.00526-25-s0007.tif]
